# Association between prenatal or early postnatal exposure to perfluoroalkyl substances and language development in 18 to 36-month-old children from the Odense Child Cohort

**DOI:** 10.1186/s12940-023-00993-w

**Published:** 2023-05-30

**Authors:** Iben Have Beck, Niels Bilenberg, Helle Raun Andersen, Fabio Trecca, Dorthe Bleses, Tina Kold Jensen

**Affiliations:** 1 Department of Clinical Pharmacology, Pharmacy and Environmental Medicine, Institute of Public Health, University of Southern Denmark, J.B. Winsløwsvej 17A, 2, Odense, 5000 Denmark; 2 Department of Child and Adolescent Mental Health Odense, Mental Health Services in the Region of Southern Denmark, Odense, Denmark; 3 Odense Patient data Explorative Network (OPEN), Odense, Denmark; 4 Hans Christian Andersen Children’s Hospital, Odense University Hospital, Odense, Denmark; 5 TrygFonden’s Centre for Child Research and School of Communication and Culture, Aarhus, Denmark

**Keywords:** Perfluoroalkyl substances, PFAS, Neurodevelopment, Language development, MacArthur-Bates Communicative Development inventories, MB-CDI, The Odense Child Cohort

## Abstract

**Background:**

Perfluoroalkyl substances (PFAS) are persistent chemicals used in everyday consumer products leading to ubiquitous human exposure. Findings of impaired neurodevelopment after prenatal exposure to PFAS are contradictory and few studies have assessed the impact of postnatal PFAS exposure. Language development is a good early marker of neurodevelopment but only few studies have investigated this outcome separately. We therefore investigated the association between prenatal and early postnatal PFAS exposure and delayed language development in 18 to 36-month-old Danish children.

**Methods:**

The Odense Child Cohort is a large prospective cohort. From 2010 to 2012 all newly pregnant women residing in the Municipality of Odense, Denmark was invited to participate. Concentration of perfluorooctane sulfonic acid (PFOS), perfluorooctanoic acid (PFOA), perfluorohexane sulfonic acid (PFHxS), perfluorononanoic acid (PFNA) and perfluorodecanoic acid (PFDA) were assessed in maternal serum collected in the 1st trimester of pregnancy and in child serum at 18 months. Parents responded to the Danish adaption of the MacArthur-Bates Communicative Development Inventories (MB-CDI) when their child was between 18 and 36 months. Language scores were converted into sex and age specific percentile scores and dichotomized to represent language scores above or below the 15th percentile. We applied Multiple Imputation by Chained Equation and conducted logistic regressions investigating the association between prenatal and early postnatal PFAS exposure and language development adjusting for maternal age, pre-pregnancy BMI, education and respectively fish intake in pregnancy or childhood and duration of breastfeeding in early postnatal PFAS exposure models.

**Results:**

We found no significant associations between neither prenatal nor early postnatal PFAS exposure and language development among 999 mother-child pairs.

**Conclusion:**

In this low-exposed cohort the finding of no association between early postnatal PFAS exposure and language development should be interpreted with caution as we were unable to separate the potential adverse effect of PFAS exposure from the well documented positive effect of breastfeeding on neurodevelopment. We, therefore, recommend assessment of child serum PFAS at an older age as development of the brain proceeds through childhood and even a small impact of PFAS on neurodevelopment would be of public health concern at population level due to the ubiquitous human exposure.

**Supplementary Information:**

The online version contains supplementary material available at 10.1186/s12940-023-00993-w.

## Background

Perfluoroalkyl substances (PFAS) are persistent chemicals widely used in the industrial and commercial production of water-resistant or repellent fabrics, non-stick coatings, grease proof materials and firefighting foams [1]. Humans are exposed to these substances in everyday life through ingestion of contaminated drinking water and food and inhalation of dust [2, 3]. PFAS are detectable in almost all human serum-samples [4, 5]. Legacy PFAS including perfluorooctane sulfonic acid (PFOS) and perfluorooctanoic acid (PFOA) have been restricted under the Stockholm Convention [6]. However, due to their long elimination half-lives [7] they are still the most frequently detected PFAS in humans [8, 9].

PFAS cross the placenta and expose the developing foetus [10, 11]. The foetal and young child brain is particularly vulnerable to exposure to neurotoxicants due to the rapid growth and complex development [12]. Exposure to PFAS during these critical time windows may have long term impact on neurodevelopment in children [11, 13, 14]. Prenatal exposure to PFOA and PFOS has been found to cause disturbance in neurodevelopment in rodents [15–20]. A recent study from the Odense Child Cohort (OCC) suggested that prenatal exposure to PFOS and PFNA adversely affected neurodevelopment as measured by IQ at age 7 years [21]. However, as competing exposures potentially could affect neurodevelopment at age 7 years it is of relevance to investigate associations between prenatal and early postnatal PFAS exposure and neurodevelopment in younger children.

Language development is regarded a reliable proxy for neurodevelopment in toddlers [22]. Evidently, language development cannot be assessed in animal models and studies with assessment of language development as an independent outcome are limited and findings are contradictory [23–31]. Language development in toddlers predict academic achievement in school children [32, 33] and the European Food Safety Authority (EFSA) has concluded that evidence on prenatal PFAS exposure and adverse neurodevelopmental outcomes in humans including language development is insufficient [6]. Neurodevelopment take place throughout childhood [14] and early postnatal exposure to PFAS could be as detrimental as prenatal exposure. To our knowledge, no studies have investigated the association between early postnatal PFAS exposure and language development in toddlers. We therefore aimed to investigate if prenatal or early postnatal PFAS exposure was associated with language development in 18 to 36-month-old Danish children.

## Methods

### Study sample and setting

The Odense Child Cohort is an on-going prospective birth cohort. Between 2010 and 2012 all newly pregnant women (gestational week (GW) < 16) living in the Municipality of Odense (Denmark) were invited to take part in the study [34]. They received information and were invited to donate a blood sample at inclusion (GW 8–16). Following enrolment participants responded to a questionnaire about general health and lifestyle including maternal education level (high school or less; high school + 1–4 years; high school + > 4 years), height, weight used for calculating pre-pregnancy body mass index (BMI) kg/m^2^ and average dietary fish intake for lunch or dinner (never, 0–3 times or 4–7 times per week), as fatty acids found in fish have been positively associated with brain growth and development [35–38]. At the same time fish diet is a significant source to PFAS exposure [1]. Data on maternal age at delivery (< 28, 28–34 or > 34 years) and birth characteristics including preterm birth (born before GW 37) were collected from obstetric and paediatric hospital records. Information on child health, including total duration of breastfeeding (continuous in months), was obtained from questionnaires filled in by parents at age 3 and 18 months. Information about duration of breastfeeding of older siblings was not available. From the questionnaire, we further attained information about day care attendance and dietary fish intake from age 12 to 18 months (never/hardly ever, weekly or daily).[39].

### PFAS analyses

PFAS analyses were conducted at the Department of Environmental Medicine, SDU, using on-line solid-phase extraction followed by liquid chromatography and triple quadruple mass spectrometry (LC-MS/MS) [39]. The quantification of PFAS included: perfluorooctane sulfonic acid (PFOS), perfluorooctanoic acid (PFOA), perfluorohexane sulfonic acid (PFHxS), perfluorononanoic acid (PFNA) and perfluorodecanoic acid (PFDA).

All available serum samples collected from mothers during pregnancy and from 18-month-old children have been analyzed for PFAS. Before exclusion of miscarriages stillbirths, drop-outs, and multiple pregnancies 200 maternal samples (GW 8–16) were selected at random and analyzed in 2011 [39]. Based on availability of data from birth records, questionnaires, and clinical examinations at age 3 months, another 449 samples were analyzed in 2013-14 [40] and the remaining 979 maternal samples were analyzed in 2019. Children’s samples collected at 18 months of age (n = 941) were all analyzed in 2020 [41]. The within-batch coefficients of variation (CVs) were < 3% and the between batch CVs for all sets were < 10.5%. Quality control and quality assessment were based on certified reference material (NIST1958) from the National Institute of Standards and Technology [39, 41]. Further information on the analytical methods have previously been described [21, 39, 41]. PFOS, PFOA, PFHxS, PFNA and PFDA were measurable in all samples except for 7 (≈ 0.5%) maternal samples with PFHxS concentrations below limit of quantification (LOQ (0.03 ng/mL)). As we were able to quantify PFAS down to the same level as limit of detection (LOD) (0.03 ng/mL), the 7 samples below LOQ were assigned LOQ/2 in the statistical analyses [39].

**Fig. 1:**
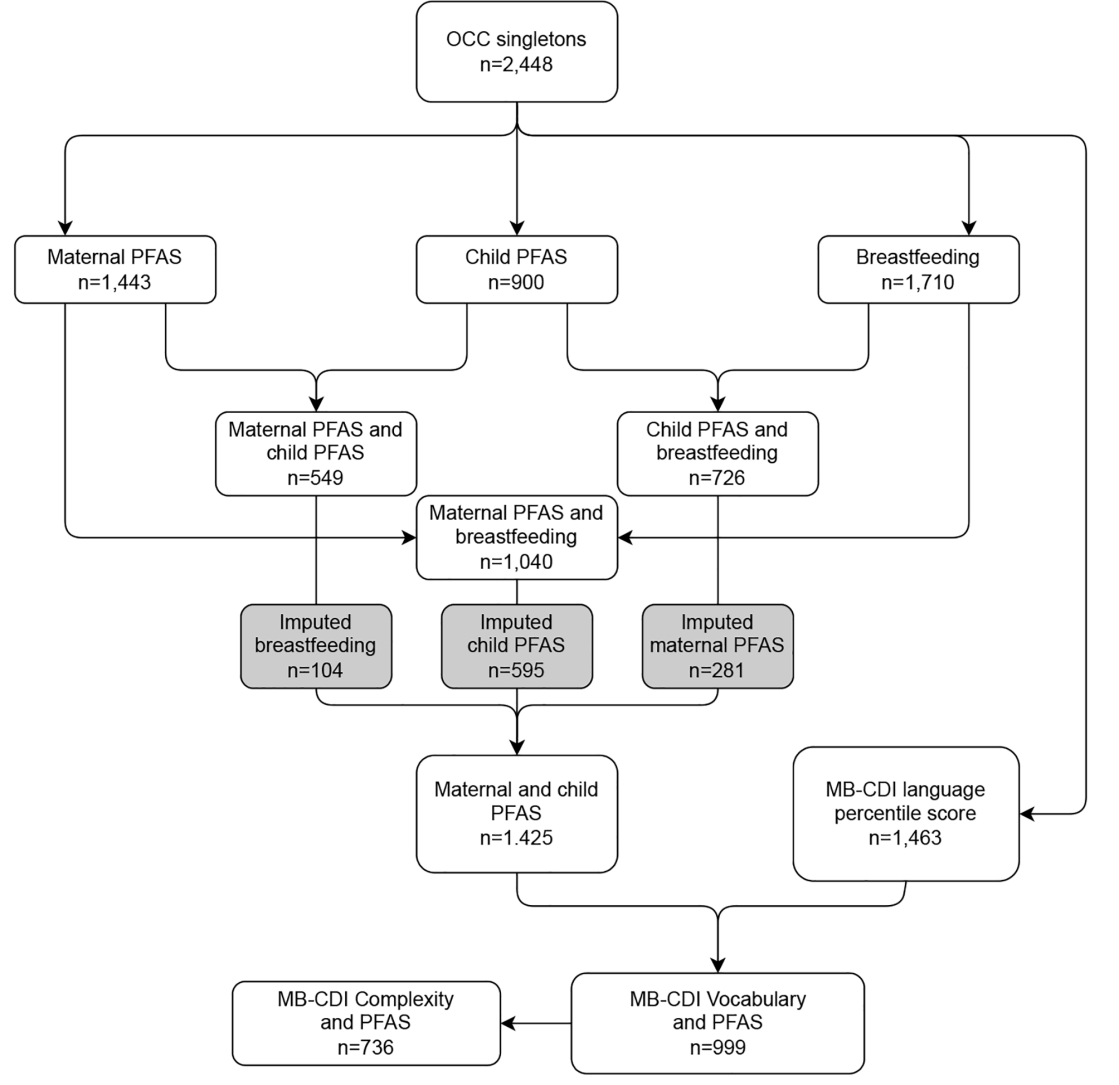
Flowchart. Selection of study sample based on data availability of language scores and availability of two out of three variables: maternal PFAS, child PFAS and/or duration of breastfeeding. Grey boxes indicate number of values imputed using Multiple Imputation by Chained Equations (MICE).

For this study maternal serum PFAS concentrations were available from 1,443 mothers of singletons and child serum PFAS concentrations from 900 singletons (Fig. 1) [40][21].

### Assessment of language development

Language development was assessed using the validated Danish adaptation [42] of the MacArthur-Bates Communicative Development Inventories (MB-CDI), which is designed to capture parents’ knowledge of their child’s language skills [43]. Every third month from age 18 to 36 months parents were encouraged to complete an electronic version of the MB-CDI Words and Sentences (MB-CDI: WS), which is normed for toddlers from 16 months of age. The MB-CDI: WS addresses seven language domains. We selected two domains representing important aspects of language development at this particular age span, which also are known to predict future reading abilities [32]. The first language domain, *Vocabulary*, focuses on the child´s production of commonly used words for toddlers (lexicon) and contains 725 words belonging to 22 semantic categories (e.g., *animals, clothing, vehicles*). The second language domain, focuses on the child´s use of grammar and syntactic complex sentences (morphosyntax) and contains 33 items (e.g., *Two car* vs. *Two cars*, *No wash the doll* vs. *Don’t wash the doll*). From 18 to 36 months of age productive vocabulary summary scores (number of correctly produced words) was generated, whereas, the complexity summary scores (number of produced complexity items), started from age 30 months for boys and age 26 months for girls, as more than 15% of the children scored zero before these ages [42]. Furthermore, as a large proportion of the parents only completed a single (5%), two (16%) or three (15%) questionnaires we selected data from the first MB-CDI: WS questionnaire completed for each child. In these vocabulary summary scores were available in a total of 1,463 children of which 1,051 also had complexity summary scores (Fig. 1). Children were on average 21 months old (IQR (21;24); range (20-26)) at vocabulary assessment and 30 months old (IQR (27;32); range (26-36)) at complexity assessment. To facilitate comparison across child sex and age at assessment each child was assigned sex and age specific percentile language scores according to the Danish MB-CDI reference study, which included 3,714 Danish children aged 16 to 36-months [42]. This procedure resulted in two separate language percentile scores (vocabulary and complexity) for each child. Vocabulary and complexity percentile scores were dichotomized ≤ or > the 15th percentile in accordance with the reference study, where a percentile score ≤ 15 was defined as delayed language development [42]. In the reference study, children whose parents had different ethnicity (indicating upbringing in a bilingual family), whose parents were living apart, or who suffered from any chronic disease or speech/speech-hearing problems were excluded [42]. We did not obtain information about these factors in this study and were therefore not able to exclude these children.

### Statistical analyses

**Fig. 2:**
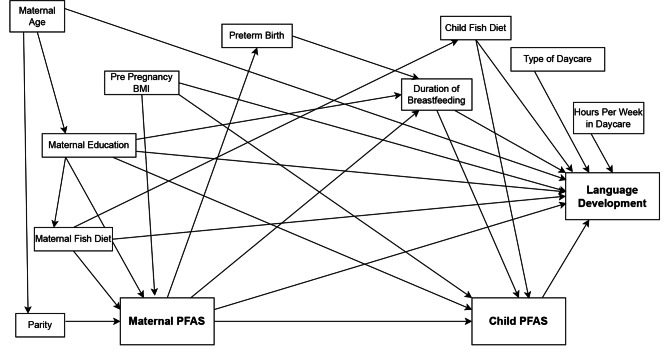
Directed Acyclic Graph (DAG). Presentation of hypothesized paths between exposures and outcomes including potential confounders, mediators, and strong predictors

We defined our study sample based on availability of language scores and of two of following three variables; maternal PFAS, child PFAS and/or duration of breastfeeding as maternal serum PFAS concentrations and duration of breastfeeding were assumed to predict child serum PFAS concentrations measured at 18 months and vice versa in a multiple imputation model (Fig. 1)[21]. Potential confounders associated with language development/neurodevelopment were selected a-priori based on existing literature [44, 45][35–38] (Fig. 2).

Due to missing values, we used Multivariate Imputation by Chained Equations (MICE) generating 50 datasets with 10 iterations each [46]. Missing values in maternal PFAS (missing: 20%), child PFAS (41%), maternal education (1%), maternal fish intake (33%), preterm birth (< 1%), birthweight (< 1%), duration of breastfeeding (6%), child fish intake (12%), type of day care (12%) and hours per week in day care (11%) were imputed using non-missing values and values from variables with no missingness including maternal age, pre pregnancy BMI, parity, child sex and language scores as well as available anthropometric measures from birth and child examination at child age 18 months and year of inclusion as auxiliary variables.

We compared categorical variables (maternal education, pre-pregnancy BMI, age, parity, sex, birthweight z-score and duration of breastfeeding) among our study sample and non-participants using chi-squared test. We examined correlations between maternal and child PFAS concentrations and their correlation to duration of breastfeeding using Spearman’s rank correlation coefficient (Spearman’s rho).

Differences between both dichotomized language percentile scores and median maternal and child serum PFAS concentrations were investigated according to categorical maternal and child characteristics using Chi-squared test and Kruskal-Wallis or Mann-Whitney test.

PFAS concentrations were transformed using the natural logarithm (ln) due to skewed distribution. We conducted simple and multiple logistic regressions between ln-PFAS concentrations and dichotomized language scores. Regressions on maternal serum PFAS, including prenatal PFOS, PFOA, PFHxS, PFNA and PFDA exposure were adjusted for maternal age, education, pre-pregnancy BMI, and fish intake during pregnancy, whereas regressions on child serum PFAS, including early postnatal PFOS, PFOA, PFHxS, PFNA and PFDA exposure were adjusted for maternal age, education, pre-pregnancy BMI, duration of breastfeeding and child fish intake at age 18 months. Estimates were back transformed to represent odds ratio (OR) of having a language score (vocabulary or complexity) below the 15th percentile, per doubling of PFAS exposure (ng/mL).

We conducted one sensitivity analysis excluding younger siblings included in the study to test for potential data dependency and another excluding preterm born children, as preterm birth could be associated with delayed language development at this young age. Lastly, we tested the effect of adjustment for duration of breastfeeding by omitting duration of breastfeeding from the early postnatal PFAS exposure models.

Estimates were presented with 95% confidence intervals (CI) and p-values < 0.05 were considered statistically significant. Statistical analyses were conducted in Stata/IC version 17.0.

## Results

A total of 2,448 singleton mother-child pairs were enrolled in the OCC. From this group 1,425 had data on two out of three defining variables (maternal PFAS, child PFAS and/or duration of breastfeeding) and were thus considered eligible for MICE (see above). From this sample vocabulary percentile scores were available in a total of 999 children, and 736 children also had complexity percentile scores (Fig. 1). Compared to non-participants (n = 1,449), the participating mothers (n = 999) were more often primipara, overweight, had longer education and breastfed their child for a longer period. No differences in maternal age, child sex or birth weight between participants and non-participants were found (Additional Table 1).

Median PFOS, PFOA, PFHxS, PFNA and PFDA (ng/mL) were 7.84, 1.70, 0.36, 0.65 and 0.29 in maternal serum and 4.80, 2.48, 0.34, 0.58 and 0.18 in child serum, respectively (Additional Table 2). Children were breastfed for median (IQR) 7.4 (3.7;10.6) months. Moderate correlations between maternal and child concentrations were positive for all PFAS (Table 1). Maternal PFAS was weakly inversely correlated with duration of breastfeeding, whereas stronger positive correlations were observed between duration of breastfeeding and child PFAS (Table 1). Higher maternal PFAS were observed among younger, primiparous mothers with shorter education. Child PFAS concentrations were higher among firstborn children of older mothers with long education and in children breastfed for more than 3 months (Additional Table 2).

**Table 1:** Spearman’s rank correlation coefficient (Spearman’s ρ) between maternal (Ma) PFAS, child (Ch) PFAS and total duration of breastfeeding (BF) in 999 singleton mother-child pairs in the Odense Child cohort

	MaPFOS	MaPFOA	MaPFHxS	MaPFNA	MaPFDA	ChPFOS	ChPFOA	ChPFHxS	ChPFNA	ChPFDA	BF
MaPFOS	1.00										
MaPFOA	0.59*	1.00									
MaPFHxS	0.53*	0.45*	1.00								
MaPFNA	0.62*	0.65*	0.44*	1.00							
MaPFDA	0.40*	0.42*	0.28*	0.62*	1.00						
ChPFOS	0.35*	0.20*	0.28*	0.28*	0.21*	1.00					
ChPFOA	0.19*	0.40*	0.22*	0.34*	0.26*	0.75*	1.00				
ChPFHxS	0.20*	0.14*	0.31*	0.16*	0.14*	0.86*	0.74*	1.00			
ChPFNA	0.14*	0.16*	0.15*	0.30*	0.30*	0.80*	0.75*	0.72*	1.00		
ChPFDA	0.13*	0.15*	0.12*	0.22*	0.27*	0.63*	0.60*	0.52*	0.80*	1.00	
BF	-0.14*	-0.14*	-0.02	-0.08*	0.02	0.59*	0.60*	0.61*	0.55*	0.40*	1.00

*p < 0.05 using Spearman’s rank correlation test

Abbreviations: PFOS, perfluorooctane sulfonic acid; PFOA, perfluorooctanoic acid; PFHxS, perfluorohexane sulfonic acid; PFNA, perfluorononanoic acid; PFDA, perfluorodecanoic acid; Ma, maternal; Ch, child; BF, total duration of breastfeeding

After conversion into sex and age specific language percentile scores the median (IQR) vocabulary and complexity percentile scores were 50 (25;75) and 45 (15;70), respectively. Median child age at MB-CDI Vocabulary assessment was 21 months (IQR (21;23); range (20-36)) at vocabulary assessment and 30 months old (IQR (27;32); range (26-36)) at complexity assessment (data not shown). A total of 181 (18%) and 189 (26%) children had complexity and vocabulary scores ≤ the 15th percentile, respectively (Table 2). No age differences at assessment time were seen between children with delayed and normal language development (data not shown).

Mothers of children with low language percentile scores were older, had shorter education, higher BMI and reported lower intake of fish during pregnancy and in their children’s diet and shorter duration of breastfeeding as compared to mothers of children with language scores above the 15th percentile (Table 2).

**Table 2:** Distribution of characteristics according to language scores ≤ and > the 15th percentile in 999 mother-child pairs from the Odense Child Cohort, Odense, Denmark

Maternal and child characteristics	MB-CDI vocabulary percentile score			MB-CDI complexity percentile score		
	All (n = 999) %	≤ 15 (n = 181) %	> 15 (n = 818) %	All (n = 736) %	≤ 15 (n = 189) %	> 15 (n = 547) %
**Educational level**						
Short	26	31	25	26	28	26
Intermediate	52	50	52	51	52	50
Long	22	19	23	23	20	24
**BMI 3 (kg/m** ^**2**^ **)**						
< 25 (under/normal)	64	59	65	61	57	63
25–30 (overweight)	26	30	25	27	34	25
> 30 (obese)	10	11	10	12	9	12
**Fish diet**						
Never	12	12	13	12	20*	11*
0–3 times/week	82	85	81	82	75*	84*
4–7 times/week	6	3	6	6	6*	5*
**Parity**						
1	57	55	57	57	57	56
2	34	33	35	34	33	35
2+	9	12	8	9	10	9
**Age (years)**						
< 28	25	22	26	24	19*	25*
28–34	50	46	50	51	46*	53*
> 34	25	32	24	25	35*	22*
**Sex**						
Boy	54	57	53	50	53	49
Girl	46	43	47	50	47	51
**Preterm (GW < 37)**						
Yes	2	2	2	2	3	1
No	98	98	98	98	97	99
**z-Birthweight**						
1st quartile	26	25	26	24	23	24
2nd -3rd quartile	47	45	48	49	51	48
4th quartile	27	30	26	27	26	28
**Day care (DC)**						
Nursery/day care	73	75	72	74	76	73
Integrated inst.	27	25	28	26	24	27
**Hours/week in DC**						
< 30 hours	17	15	17	15	14	15
30–35 hours	36	39	35	37	37	37
> 35 hours	47	46	48	48	49	48
**Total breastfeeding**						
≤ 3 months	21	28*	20*	21	25	19
> 3 months	79	72*	80*	79	75	81
**Fish diet at 18 months**						
Never/hardly ever	23	34*	22*	22	31	20
Weekly	47	41*	46*	48	39	48
Daily	30	25*	32*	30	30	31
**Educational level**						
Short	26	31	25	26	28	26
Intermediate	52	50	52	51	52	50
Long	22	19	23	23	20	24
**BMI 3 (kg/m** ^**2**^ **)**						
< 25 (under/normal)	64	59	65	61	57	63
25–30 (overweight)	26	30	25	27	34	25
> 30 (obese)	10	11	10	12	9	12
**Fish diet**						
Never	12	12	13	12	20*	11*
0–3 times/week	82	85	81	82	75*	84*
4–7 times/week	6	3	6	6	6*	5*

*p < 0.05 using Chi-squared test.

Abbreviations: BMI, body mass index; DC, day care; MB-CDI, MacArthur-Bates Communicative Development Inventories

In logistic regression analysis we found no significant associations between prenatal or early postnatal PFAS exposure and language development.

**Fig. 3:**
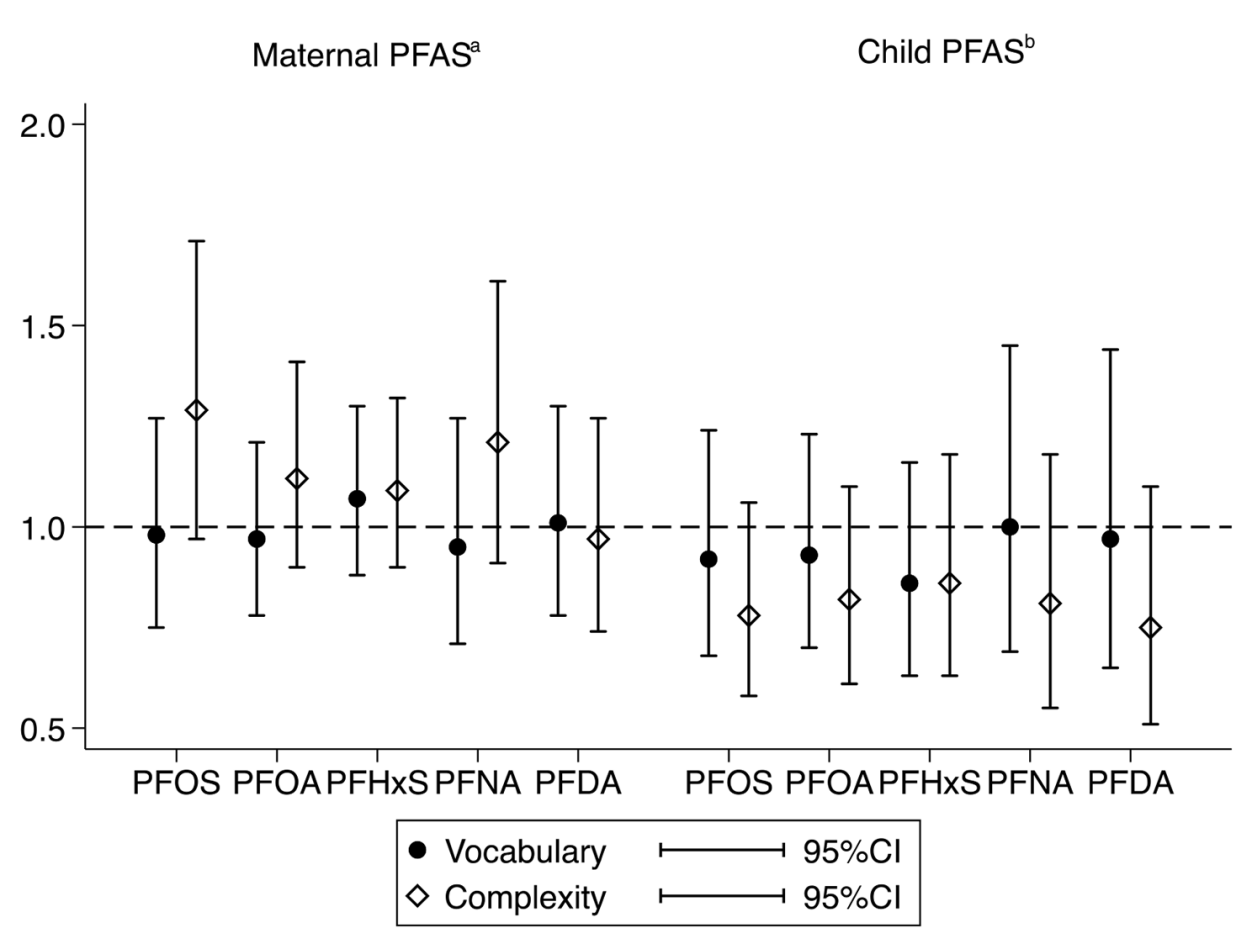
Odds ratios (OR) and 95% confidence intervals (95% CI) for sex and age specific Vocabulary and Complexity scores below the 15th percentile for a doubling in prenatal and early postnatal PFAS exposure, respectively a) adjusted for maternal education, pre-pregnancy BMI, age, and fish diet b) adjusted for maternal education, pre-pregnancy BMI, age, duration of breastfeeding and child fish diet Abbreviations: PFAS, perfluoroalkyl substances; PFOS, perfluorooctane sulfonic acid; PFOA, perfluorooctanoic acid; PFHxS, perfluorohexane sulfonic acid; PFNA, perfluorononanoic acid; PFDA, perfluorodecanoic acid; CI, confidence interval

The ORs for associations between a doubling in prenatal PFAS exposure and Vocabulary and Complexity percentile scores ≤ 15 were likewise close to 1 in crude models (Additional Table 3) and after adjustment for maternal age, education, pre-pregnancy BMI, and fish intake during pregnancy (Fig. 3).

ORs for Vocabulary and Complexity percentile scores ≤ 15 after a doubling in early postnatal PFAS exposure were also close to 1 and not statistically significant after adjustment for maternal age, education, pre-pregnancy BMI, duration of breastfeeding and child fish intake at age 18 months (Fig. 3).

Exclusion of the younger siblings (n = 18) or preterm children (n = 15) did not change the direction or magnitude of estimates (data not shown). Exclusion of duration of breastfeeding in analyses of early postnatal PFAS exposure did not change the direction or magnitude of the estimates (Additional Table 4).

## Discussion

We found no statistically significant associations between neither prenatal nor early postnatal PFAS exposure and language development (vocabulary and complexity) in 18 to 36-month-old children from the Odense Child Cohort. A previous study within the cohort found association between higher maternal PFAS exposure and lower Full Scale IQ in the offspring at age 7 years [21]. Interestingly, the findings between PFAS exposure and verbal sub-domains of IQ were less pronounced than those of non-verbal sub-domains. This finding, together with the results of the present study, may indicate that language development is less susceptible to PFAS exposure than other distinct domains of brain development.

Language development is believed to commence in the central nervous system assessed from GW 31 through readiness for sound [47] and speech processing [48]. The last trimester of pregnancy could therefore be a susceptible window of vulnerability for exposure to neurotoxic substances with potential adverse effects on language development. The mechanisms behind the potential effects of PFAS on neurodevelopment are not fully understood, however, different experimental models investigating prenatal PFAS exposure in rodents suggest clear indication of neurotoxicity through alterations of the calcium homeostasis, synaptic plasticity, cellular differentiation and disturbance of thyroid hormone function [16, 17, 19, 20].

Only one previous study has to our knowledge investigated associations between prenatal PFAS exposure and language development as a main outcome among 448 British mother-daughter pairs from the Avon Longitudinal Study of Parents and Children (ALSPAC) cohort [26]. Despite generally higher maternal PFOS, PFOA, PFHxS and PFNA serum concentrations as compared to pregnant women in the OCC (19.8, 3.7, 1.9 and 0.5 vs. 7.8, 1.7, 0.4 and 0.7 ng/mL), the ALSPAC study reported no significant associations between maternal serum PFAS measured in 1st trimester of pregnancy and language development measured by MB-CDI in 15 and 38-month-old girls [26], which is in accordance with our findings.

Other studies have investigated the association between prenatal PFAS exposure and language development as a sub-domain of overall neurodevelopment, resulting in less elaborated descriptions and interpretations of null- or non-significant findings. Generally, no associations between prenatal PFAS exposure and verbal subscales of neurodevelopmental tests have been reported among 6-month-old to 3.5-year-old children [23–25, 27–31], although some studies found tendencies of associations between higher prenatal PFAS exposure and higher scores [27, 29], and others to lower scores [23, 25, 30]. A recent study from the Shanghai Birth Cohort, however, found significant associations between prenatal PFAS exposure and lower language scores assessed as a subdomain in 2-year-old children using the Bayley Scales of Infant and Toddler Development- Third edition (BSID-III) [49]. The BSID-III is a standardized test administered by psychologists and combines a receptive and an expressive scale when generating the score for the language domain, whereas the MB-CDI subdomains used in our study were based exclusively on expressive language development which has been considered a more reliable measure [50]. However, the MB-CDI is based on parental reporting, which may introduce an additional source of unsystematic variance. Moreover, the children in the Shanghai Birth Cohort were exposed to up to 5 times higher PFNA and PFDA compared to children in our cohort, which also could explain the different findings.

To our knowledge, no studies have investigated early postnatal PFAS exposure and language development in toddlers. Serum PFAS concentrations measured at 18 months of age have previously been found to predict duration of breastfeeding in our study population [21], which could complicate the separation of a potential adverse effect of early postnatal PFAS exposure from the well documented positive effect of breastfeeding on neurodevelopment [51, 52]. This was reflected in the barely notable changes in estimates between early postnatal PFAS exposure and language scores in analysis with or without adjustment for duration of breastfeeding. The findings of no association between early postnatal PFAS exposure and language development should therefore be interpreted with caution.

Our study has several strengths, it is embedded in a large population-based cohort study, the Odense Child Cohort, where detailed information about lifestyle and behavioural factors have been collected from verification of pregnancy through birth and childhood, facilitating a unique opportunity to account for relevant confounders. Another strength is the use of the international validated language assessment tool MB-CDI, which in the Danish version is standardized according to a large Danish reference population [42, 44]. The MB-CDI also account for the well-known sex-specific differences in language development at this young age [53], as sex and age specific percentile scores were calculated.

There are, however, some limitations to our study. The sample may be prone to selection bias, as participants were more often primipara, overweight, had longer education and breastfed their child for a longer period compared to non-participants. However, as participants were not aware of their own nor their child’s PFAS exposure at enrolment, and because we compare children across PFAS exposure levels, it is of less importance whether they represent the general population. Children’s language skills were parent reported, which may lead to misclassification, although likely to be non-differential, as parents were unaware of their PFAS exposure. Hence potential bias would be towards the null. Additionally, some studies indicate that more highly educated parents tend to underestimate their children’s linguistic abilities and vice versa (Dunning-Kruger effect) [54]. As high education was associated with higher PFAS exposure in the children, this may also bias estimates towards the null. Therefore, our null findings may be due to non-differential misclassification.

Not all expecting mothers and 18-month-old children donated blood samples and we do not know whether their PFAS exposure differ from those who donated blood. However, we were able to impute missing values in exposures based on availability of two of following three variables: maternal PFAS, child PFAS and/or duration of breastfeeding, as child serum PFAS at this young age is mainly determined by prenatal exposure via placental transfer and postnatal exposure through breastfeeding.

We investigated multiple exposures. Although we did not find any significant associations, methods for handling multi-pollutant exposures could be relevant to apply in future investigations.

Lastly, we cannot rule out residual confounding from unmeasured health, lifestyle, and behavioural factors. Information on breastfeeding was self-reported and therefore less accurate, yet closely correlated to PFAS concentrations at age 18 months. Furthermore, we did not obtain neurodevelopmental endpoints for parents, nor did we obtain information about chronic disease or speech/speech-hearing problems in the children or whether they were brought up in a bilingual family.

## Conclusion

In this low-exposed cohort, we found no association between neither prenatal nor early postnatal PFAS exposure and MB-CDI assessed language development in young toddlers. The majority of similar, previous conducted studies did not find adverse associations between prenatal PFAS exposure and language development. However, our null findings should be interpreted with caution as we were not able to separate the potential adverse effect of PFAS exposure from the well documented positive effect of breastfeeding on neurodevelopment. Furthermore, we cannot rule out any adverse associations at higher exposure levels. As PFAS exposure is ubiquitous, even a minor impact of PFAS on neurodevelopment could be a public health burden at population level. Therefore, we recommend assessment of additional neurodevelopmental endpoints including non-verbal domains, as well as measuring PFAS concentrations at an older age, enabling separation of the potential adverse impact of childhood PFAS exposure from the beneficial effect of breastfeeding on neuropsychological development.

## Electronic supplementary material

Below is the link to the electronic supplementary material.

Additional Table 1. Distribution of characteristics in 2,448 active participants, presented in the current study sample (n = 999) and the remaining participants in the Odense Child Cohort (n = 1,449), Denmark.

Additional Table 2. Median maternal and child PFAS concentrations (ng/mL) according to maternal and child characteristics in 999 mother-child pairs from the Odense Child Cohort, Odense, Denmark.

Additional Table 3. Crude and adjusted odds ratios (OR) and 95% confidence intervals (95% CI) for sex and age specific Vocabulary and Complexity scores below the 15th percentile when prenatal and early postnatal PFAS exposure are doubled, in 999 children from Odense Child Cohort.

Additional Table 4. Adjusted Odds ratios (OR) and 95% confidence intervals (95% CI) for sex and age specific Vocabulary and Complexity scores below the 15th percentile when early postnatal PFAS exposure are doubled, in 999 children from Odense Child Cohort without adjustment for duration of breastfeeding.

## Data Availability

The datasets used and analysed during the current study are available from the corresponding author on reasonable request.

## List of Abbreviations

ALSPAC: Avon Longitudinal Study of Parents and Children
BMI: Body mass index
BSID-III: Bayley Scales of Infant and Toddler Development- Third edition
CI: Confidence interval
EFSA: European Food Safety Authority
GW: Gestational week
IQR: Interquartile range
LC-MS/MS: Liquid chromatography and triple quadruple mass spectrometry
Ln: Natural logarithm
MB-CDI: MacArthur-Bates Communicative Development Inventories
MICE: Multivariate Imputation by Chained Equations
mL: Millilitres
ng: Nano grams
OCC: Odense Child Cohort
OPEN: Odense Patient data Explorative Network
PFAS: Perfluoroalkyl substances
PFDA: Perfluorodecanoic acid
PFHxS: Perfluorohexane sulfonic acid
PFNA: Perfluorononanoic acid
PFOA: Perfluorooctanoic acid
PFOS: Perfluorooctane sulfonic acid
