# Supplementary material for: Association between prenatal or early postnatal exposure to perfluoroalkyl substances and language development in 18 to 36-month-old children from the Odense Child Cohort
Source: Environ Health. 2023 May 30;22:46. doi: 10.1186/s12940-023-00993-w (PMC10228033; doi:10.1186/s12940-023-00993-w)
Supplement: Supplementary file 1 — Additional Table 1. Distribution of characteristics in 2,448 active participants, presented in the current study sample (n = 999) and the remaining participants in the Odense Child Cohort (n = 1,449), Denmark. [file 12940_2023_993_MOESM1_ESM.docx]

Additional table 1. Distribution of characteristics in 2,448 active participants, presented in the current study sample (n=999) and the remaining participants in the Odense Child Cohort (n=1,449), Denmark.

|  | All  n=2,448 | Study pop  n=999 | Non-participants n=1,449 |
| --- | --- | --- | --- |
|  | % (n) | % (n) | % (n) |
| Maternal characteristics | |  | |
| Age (years)  <28  28-34  >34 | 27% (671)  49% (1,200)  23% (577) | 25% (251)  50% (496)  25% (252) | 29% (420)  49% (704)  22% (325) |
| BMI 3 (kg/m^2^)  <25  25-30  >30  Missing | 66% (1,620)  23% (556)  11% (271)  (1) | 63% (634)*  26% (261)*  11% (104)* | 68% (986)*  20% (295)*  12% (167)*  (1) |
| Education level  High school or less  High school +1-4 y  High school +>4 y  Missing | 30% (725)  49% (1,171)  21% (492)  (60) | 26% (258)*  52% (509)*  22% (218)*  (14) | 33% (467)*  47% (662)*  20% (274)*  (46) |
| Parity  1  2  2+ | 55% (1,351)  34% (829)  11% (268) | 57% (568)*  34% (341)*  9% (90)* | 54% (783)*  34% (488)*  12% (178)* |
| Child characteristics |  |  | |
| Sex  Boy  Girl | 53% (1,293)  47% (1,154) | 54% (539)  46% (460) | 52% (754)  48% (695) |
| Birthweight z-score  1^st^ quartile  2^nd^-3^rd^ quartile  4^th^ quartile  Missing | 25% (609)  50% (1,217)  25% (608)  (14) | 26% (255)  47% (472)  27% (268)  (4) | 24% (354)  52% (745)  24% (340)  (10) |
| Breastfeeding  ≤ 3 months  >3-≤12 months  Missing | 25% (429)  75% (1,281)  (738) | 22% (207)*  78% (735)*  (57) | 29% (222)*  71% (546)*  (681) |
| *) p<0.05 using Chi2 test | | | |
