# Supplementary material for: Association between prenatal or early postnatal exposure to perfluoroalkyl substances and language development in 18 to 36-month-old children from the Odense Child Cohort
Source: Environ Health. 2023 May 30;22:46. doi: 10.1186/s12940-023-00993-w (PMC10228033; doi:10.1186/s12940-023-00993-w)
Supplement: Supplementary file 2 — Additional Table 2. Median maternal and child PFAS concentrations (ng/mL) according to maternal and child characteristics in 999 mother-child pairs from the Odense Child Cohort, Odense, Denmark. [file 12940_2023_993_MOESM2_ESM.docx]

Additional table 2. Median maternal and child PFAS concentrations (ng/mL) according to maternal and child characteristics in 999 mother-child pairs from the Odense Child Cohort, Odense, Denmark.

| Characteristics | | Median maternal PFAS concentrations (ng/mL) | | | | | Median child PFAS concentrations (ng/mL) | | | | |
| --- | --- | --- | --- | --- | --- | --- | --- | --- | --- | --- | --- |
|  | % (n) | PFOS | PFOA | PFHxS | PFNA | PFDA | PFOS | PFOA | PFHxS | PFNA | PFDA |
| All | 100 (999) | 7.84 | 1.70 | 0.36 | 0.65 | 0.29 | 4.80 | 2.48 | 0.34 | 0.58 | 0.18 |
| **Educational level**  Short  Intermediate  Long | 26 (262)  52 (516)  22 (221) | 8.11*  7.95*  7.32* | 1.90*  1.66*  1.65* | 0.35  0.35  0.39 | 0.65  0.64  0.66 | 0.28*  0.28*  0.31* | 4.04*  4.91*  5.37* | 2.15*  2.45*  2.87* | 0.25*  0.35*  0.41* | 0.50*  0.58*  0.64* | 0.16*  0.18*  0.20* |
| **BMI 3 (kg/m^2^)**  <25 (under/normal)  25-30 (overweight)  >30 (obese) | 64 (634)  26 (261)  10 (104) | 7.92  7.82  7.41 | 1.74  1.66  1.75 | 0.37*  0.34*  0.33* | 0.67*  0.63*  0.60* | 0.30*  0.28*  0.23* | 4.99*  4.90*  3.60* | 2.56*  2.46*  1.88* | 0.36*  0.34*  0.25* | 0.61*  0.58*  0.43* | 0.18*  0.17*  0.15* |
| **Fish diet**  Never  0-3 times/week  4-7 times/week | 12 (119)  82 (815)  6 (65) | 7.04*  7.94*  7.36* | 1.72  1.70  1.81 | 0.31*  0.36*  0.46* | 0.56*  0.66*  0.79* | 0.24*  0.29*  0.31* | 3.42*  5.03*  4.12* | 2.08*  2.54*  2.57* | 0.25*  0.35*  0.32* | 0.43*  0.59*  0.62* | 0.15*  0.18*  0.17* |
| **Parity**  1  2  2+ | 57 (568)  34 (341)  9 (90) | 8.63*  7.40*  5.26* | 2.13*  1.25*  0.99* | 0.40*  0.32*  0.24* | 0.71*  0.58*  0.52* | 0.30*  0.27*  0.25* | 5.05*  4.45*  3.98* | 2.87*  2.10*  2.00* | 0.37*  0.30*  0.32* | 0.60*  0.53*  0.59* | 0.18*  0.17*  0.18* |
| **Age (years)**  <28  28-34  >34 | 25 (251)  50 (496)  25 (252) | 8.27*  8.07*  6.98* | 1.98*  1.76*  1.33* | 0.36  0.37  0.35 | 0.69*  0.65*  0.60* | 0.29*  0.30*  0.25* | 4.52  4.88  4.89 | 2.51  2.52  2.32 | 0.31*  0.34*  0.36* | 0.53*  0.59*  0.59* | 0.17*  0.18*  0.18* |
| **Sex**  Boy  Girl | 54 (539)  46 (460) | 7.87  7.74 | 1.74  1.68 | 0.37  0.34 | 0.65  0.65 | 0.29  0.29 | 4.62  4.99 | 2.49  2.48 | 0.33  0.35 | 0.59  0.57 | 0.18  0.18 |
| **Preterm (GW<37)**  Yes  No | 2 (15)  98 (984) | 7.48  7.84 | 1.70  1.70 | 0.37  0.36 | 0.68  0.65 | 0.27  0.29 | 3.59*  4.82* | 2.32  2.49 | 0.23*  0.34* | 0.45*  0.58* | 0.17  0.18 |
| **z-Birthweight**  1^st^ quartile  2^nd^-3^rd^ quartile  4^th^ quartile | 26 (257)  47 (472)  27 (270) | 8.44*  7.92*  7.27* | 1.85*  1.77*  1.46* | 0.39*  0.36*  0.32* | 0.68*  0.66*  0.60* | 0.30*  0.30*  0.27* | 5.03  4.78  4.44 | 2.70*  2.53*  2.21* | 0.37*  0.34*  0.32* | 0.61*  0.59*  0.54* | 0.18  0.18  0.17 |
| **Daycare (DC)**  Nursery/daycare  Integrated inst. | 73 (730)  27 (269) | 7.83  7.86 | 1.76  1.62 | 0.36  0.35 | 0.66  0.62 | 0.29  0.29 | 4.75  4.99 | 2.49  2.45 | 0.33  0.35 | 0.56  0.60 | 0.18  0.18 |
| **Hours/week in DC**  < 30 hours  30-35 hours  > 35 hours | 17 (172)  36 (359)  47 (468) | 7.50  7.73  8.15 | 1.66  1.70  1.72 | 0.37  0.36  0.36 | 0.64*  0.62*  0.68* | 0.28  0.29  0.29 | 4.28*  4.75*  4.92* | 2.30  2.45  2.57 | 0.34  0.31  0.36 | 0.57  0.55  0.60 | 0.16*  0.17*  0.19* |
| **Breastfeeding**  ≤3 months  >3 months | 21 (210)  79 (789) | 8.56*  7.62* | 1.79  1.68 | 0.36  0.36 | 0.68  0.64 | 0.28  0.29 | 2.46*  5.51* | 1.13*  2.87* | 0.16*  0.39* | 0.34*  0.64* | 0.13*  0.19* |
| **Fish diet at 18 mo**  Never/hardly ever  Weekly  Daily | 23 (234)  46 (463)  30 (302) | 7.39*  7.93*  7.98* | 1.63  1.76  1.70 | 0.32*  0.37*  0.38* | 0.59*  0.66*  0.69* | 0.26*  0.28*  0.31* | 3.81*  4.92*  5.32* | 2.11*  2.57*  2.63* | 0.26*  0.36*  0.36* | 0.47*  0.59*  0.63* | 0.16*  0.18*  0.19* |
| Abbreviations: PFOS, Perfluorooctane sulfonic acid; PFOA, Perfluorooctanoic acid; PFHxS, Perfluorohexane sulfonic acid; PFNA, Perfluorononanoic acid; PFDA, Perfluorodecanoic acid.  *p<0.05 using Kruskal-Wallis or Mann Whitney test. | | | | | | | | | | | |
