# Supplementary material for: Association between prenatal or early postnatal exposure to perfluoroalkyl substances and language development in 18 to 36-month-old children from the Odense Child Cohort
Source: Environ Health. 2023 May 30;22:46. doi: 10.1186/s12940-023-00993-w (PMC10228033; doi:10.1186/s12940-023-00993-w)
Supplement: Supplementary file 3 — Additional Table 3. Crude and adjusted odds ratios (OR) and 95% confidence intervals (95% CI) for sex and age specific Vocabulary and Complexity scores below the 15th percentile when prenatal and early postnatal PFAS exposure are doubled, in 999 children from Odense Child Cohort. [file 12940_2023_993_MOESM3_ESM.docx]

Additional table 3. Crude and adjusted odds ratios (OR) and 95% confidence intervals (95% CI) for sex and age specific Vocabulary and Complexity scores below the 15^th^ percentile when prenatal and early postnatal PFAS exposure are doubled, in 999 children from Odense Child Cohort.

|  | **Maternal PFAS** | | **Child PFAS** | |
| --- | --- | --- | --- | --- |
|  | **MB-CDI vocabulary percentile score ≤ 15** | **MB-CDI complexity percentile score ≤ 15** | **MB-CDI vocabulary percentile score ≤ 15** | **MB-CDI complexity percentile score ≤ 15** |
|  | n ≤/> 15: 181/818 | n ≤/> 15: 189/547 | n ≤/> 15: 181/818 | n ≤/> 15: 189/547 |
|  | **Crude OR 95% CI** | | **Crude OR 95% CI** | |
| PFOS | 0.96 (0.75;1.25) | 1.20 (0.91;1.57) | 0.81 (0.65;1.00) | 0.76 (0.60;0.94)* |
| PFOA | 0.92 (0.75;1.14) | 1.04 (0.84;1.29) | 0.81 (0.67;0.99)* | 0.77 (0.63;0.95)* |
| PFHxS | 1.05 (0.86;1.27) | 1.05 (0.87;1.27) | 0.79 (0.64;0.98)* | 0.81 (0.65;1.01) |
| PFNA | 0.90 (0.69;1.19) | 1.09 (0.84;1.43) | 0.83 (0.64;1.08) | 0.76 (0.58;0.99)* |
| PFDA | 0.97 (0.76;1.24) | 0.92 (0.71;1.19) | 0.80 (0.59;1.10) | 0.70 (0.51;0.95)* |
|  | **Adjusted^a^  OR 95% CI** | | **Adjusted^b^  OR 95% CI** | |
| PFOS | 0.98 (0.75;1.27) | 1.29 (0.97;1.71) | 0.92 (0.68;1.24) | 0.78 (0.58;1.06) |
| PFOA | 0.97 (0.78;1.21) | 1.12 (0.90;1.41) | 0.93 (0.70;1.23) | 0.82 (0.61;1.10) |
| PFHxS | 1.07 (0.88;1.30) | 1.09 (0.90;1.32) | 0.86 (0.63;1.16) | 0.86 (0.63;1.18) |
| PFNA | 0.95 (0.71;1.27) | 1.21 (0.91;1.61) | 1.00 (0.69;1.45) | 0.81 (0.55;1.18) |
| PFDA | 1.01 (0.78;1.30) | 0.97 (0.74;1.27) | 0.97 (0.65;1.44) | 0.75 (0.51;1.10) |
| Abbreviations: CI, confidence interval; OR, odds ratio; PFOS, Perfluorooctane sulfonic acid; PFOA, Perfluorooctanoic acid; PFHxS, Perfluorohexane sulfonic acid; PFNA, Perfluorononanoic acid; PFDA, Perfluorodecanoic acid.  a) adjusted for maternal education, pre-pregnancy BMI, age, and fish diet  b) adjusted for maternal education, pre-pregnancy BMI, maternal age, duration of breastfeeding and child fish diet | | | | |
