# Supplementary material for: Association between prenatal or early postnatal exposure to perfluoroalkyl substances and language development in 18 to 36-month-old children from the Odense Child Cohort
Source: Environ Health. 2023 May 30;22:46. doi: 10.1186/s12940-023-00993-w (PMC10228033; doi:10.1186/s12940-023-00993-w)
Supplement: Supplementary file 4 — Additional Table 4. Adjusted Odds ratios (OR) and 95% confidence intervals (95% CI) for sex and age specific Vocabulary and Complexity scores below the 15th percentile when early postnatal PFAS exposure are doubled, in 999 children from Odense Child Cohort without adjustment for duration of breastfeeding. [file 12940_2023_993_MOESM4_ESM.docx]

Additional table 4. Adjusted Odds ratios (OR) and 95% confidence intervals (95% CI) for sex and age specific Vocabulary and Complexity scores below the 15^th^ percentile when early postnatal PFAS exposure are doubled, in 999 children from Odense Child Cohort without adjustment for duration of breastfeeding.

|  | **Child PFAS** | |
| --- | --- | --- |
|  | **MB-CDI vocabulary percentile score ≤ 15** | **MB-CDI complexity percentile score ≤ 15** |
|  | n ≤/> 15: 181/818 | n ≤/> 15: 189/547 |
|  | **Without adjustment for duration of breastfeeding^a^**  **OR 95% CI** | |
| PFOS | 0.86 (0.69;1.08) | 0.77 (0.61;0.97)* |
| PFOA | 0.87 (0.71;1.07) | 0.80 (0.64;1.00) |
| PFHxS | 0.83 (0.66;1.04) | 0.82 (0.65;1.04) |
| PFNA | 0.91 (0.68;1.21) | 0.77 (0.57;1.03) |
| PFDA | 0.90 (0.63;1.27) | 0.71 (0.50;1.01) |
| a) adjusted for maternal education, pre-pregnancy BMI, maternal age, and child fish diet. | | |
